# Does tuberculosis threaten our ageing populations?

**DOI:** 10.1186/s12879-016-1451-0

**Published:** 2016-03-11

**Authors:** Rachel Byng-Maddick, Mahdad Noursadeghi

**Affiliations:** Division of Infection and Immunity, University College London, Cruciform Building, Gower Street, London, WC1E 6BT UK

**Keywords:** Tuberculosis, Ageing, Immunosenescence

## Abstract

**Background:**

The global population is ageing quickly and our understanding of age-related changes in the immune system suggest that the elderly will have less immunological protection from active tuberculosis (TB).

**Discussion:**

Ongoing global surveillance of TB notifications shows increasing age of patients with active TB. This effect of age is compounded by changes to clinical manifestations of disease, confounding of diagnostic tests and increased rates of adverse reactions to antimicrobial treatment of TB. Future epidemiological surveillance, development of diagnostic tests and trials of treatment shortening should all include a focus on ageing people.

**Summary:**

More detailed surveillance of TB notifications in elderly people should be undertaken and carefully evaluated. Risk stratification will help target care for those in greatest need, particularly those with comorbidities or on immunosuppressive therapies. Novel diagnostics and treatment regimes should be designed specifically to be used in this cohort.

## Background

### Ageing of global populations

The global population is ageing due to longer life expectancy and lower rates of child birth. By 2035, it is projected that those aged >65 years will constitute 23 % of the total UK population [1]. Worldwide, the number of people > 60 years is expected to increase by more than double, from 841 million people in 2013 to more than two billion people in 2050 [2]. It is projected that in 2047, older persons will exceed the number of children for the first time [2]. Currently most older people live in the developed world, but the older population is also growing rapidly in less developed regions such that projections for 2050 predict nearly 80 % of the world’s older population will be living in less developed countries [2].

### Effects of age on immune protection against TB

Elderly people have an increased susceptibility to infectious diseases, particularly of the respiratory tract [3], and suffer greater morbidity and mortality compared to younger individuals [4]. Ageing has significant effects on both the innate and adaptive immune system [5–7], which may contribute to the increased risk of infection.

Immune protection against tuberculosis (TB) infection is primarily achieved by cell mediated immunity, through the coordinated action of phagocytic cells and adaptive immune T cell responses that serve to kill or contain the bacteria. In this context, macrophages represent the predominant phagocytic cells [8], CD4 T helper 1 cells are thought to enhance intracellular killing of bacteria through the action of interferon (IFN)γ and CD8 T cells may contribute to killing of infected macrophages by production of cell membrane porins such as granzyme and perforin [9].

Reduced T cell output by the thymus, T cell immune senescence, defined by reduced capacity for cell proliferation, and immune exhaustion, defined by reduced capacity to produce cytokines and other effector molecules are the most consistent reported effects of ageing on the immune system [10, 11]. In keeping with these data, ex vivo stimulation of peripheral blood mononuclear cells from elderly people with Mycobacterium tuberculosis (Mtb) antigens generates lower IFNγ responses, than in younger people [12], and older mice show increased susceptibility to Mtb infection, associated with impaired CD4 T cell-mediated immunity [13, 14]. However, others report exaggerated CD8 T cell responses in older mice which mediate a degree of increased host resistance to TB [15–17].

Age-related changes in the phenotype and function of macrophages are also reported, which might be expected to compromise their role in protection against TB. These include reduced expression of innate immune receptors, reduced production of tumour necrosis factor (TNF) and reduced phagocytosis [18]. Clear evidence for how these may impact the host-pathogen interactions between macrophages and Mtb is lacking. In one report, monocytes from older humans supported higher rates of bacterial growth [19] but others found no difference in cellular cytokine responses or bacterial growth in mouse macrophages from young and old mice [20].

The importance of well-calibrated mechanisms for regulation of inflammation to avoid or minimise the immunopathogenesis of TB are also increasingly recognised [21–23]. Persistently raised levels of proinflammatory cytokines are evident in older people [24, 25] and in pulmonary macrophages of older mice [26]. These data invite speculation that inflammage-ing may contribute to increased immunopathogenesis of TB and therefore more disease in the elderly.

## Discussion

### Evidence for the impact of ageing on TB

Currently the highest TB notification rates globally occur amongst 45–55 year olds, but in the Western Pacific Region, Eastern Mediterranean and South East Asia TB notifications are increasingly evident in older people, peaking amongst those aged ≥65 years old [27]. Similar observations have been reported in a recent review of all national TB prevalence studies in Asia which showed progressively increased TB prevalence rates amongst people over the age of 65 years [28], and this is echoed in reports from the Americas which also described the disease burden increasing amongst older adults in Central and South America [29] and North America [30].

Currently in the United Kingdom (UK), the highest incidence of TB is amongst 15–44 year olds, representing nearly 60 % of all cases and those over the age of 65 years only accounted for 14 % [31]. Interestingly, the majority of the younger cases are in non-UK born individuals, most likely due to high immigration rates in younger people from TB endemic areas, confounding our perception of TB risk in the elderly population. Even in the UK, there is a comparatively high incidence of TB in UK born individuals, greater than 75 years old [31]. Birth cohort effects, such as higher childhood or early adulthood TB transmission rates amongst the current elderly may partly explain these observations [32] as it is has long been presumed that active TB in the elderly arises from reactivation of LTBI as protective immunity wanes [33–35]. However the emerging general consensus is that LTBI is most likely to reactivate within 18 months of infection, and disease resulting from reactivation more than 10 years after infection may be rare [36] and that most active disease represents relatively recent transmission events. Whether these observations are equally true in young and old people alike is not known and merits further investigation, particularly given the age-related immune changes and co-morbidities in older people, which may increase the risk of reactivation disease.

### Effects of age on clinical presentation of TB

It has been argued that active TB infection in the elderly is “a different disease” than that occurring in younger people, due to the disparity in clinical presentation and laboratory tests [34]. Although pulmonary TB is the most common site of infection in elderly people, occurring in approximately 75 % of cases [37], many older patients with active TB may not exhibit the classic clinical features such as cough, hemoptysis, fever, night sweats and weight loss. Dyspnoea is more common and haemoptysis less common in the elderly [34, 38–40]. There may be very few symptoms, or the symptoms can mimic age-related illnesses such as reduced functional capacity, chronic fatigue, cognitive impairment, anorexia or pyrexia of unknown origin. An accurate history may be difficult to obtain due to poor memory, impaired hearing, sight or speech.

Extrapulmonary TB is more common with advancing age, and presentations may include TB meningitis, renal or bone and joint infection (particularly involving the thoracolumbar spine and large joints) [38, 41, 42]. Presentations of back and joint pain are sometimes dismissed as diseases of the elderly, such as degenerative arthritis or osteoporotic compression fractures [43], but associated constitutional symptoms (low grade fever, weight loss, fatigue and anorexia) can lead to the correct diagnosis.

### The impact of age on diagnosis

Diagnosing TB can be difficult in all age groups and is often dependent on identifying those with compatible laboratory and radiological assessments, particularly when a microbiological diagnosis may be out of reach. By comparison to the young, elderly people with TB show higher frequencies of abnormal liver enzymes, hypoalbuminaemia, hyponatraemia and hypokalaemia, and about two-thirds of elderly patients with active TB exhibit a normocytic normochromic anaemia [34]. Less pulmonary cavitation is seen on plain radiographs [37], and fewer nodules, masses and cavities on chest computed tomography have been reported in the elderly [40], albeit not always consistently [39]. Rather than isolated apical shadowing, radiographs more often show mid zone and basal shadowing often accompanied by pleural effusions [34]. Moreover, mass-like lesions or nodules due to TB in the elderly can often be difficult to distinguish from malignant disease or more common bacterial pneumonias [35].

The age-related changes to the immune system described above may also compromise immunological tests for evidence of past exposure to TB, such as the tuberculin skin test (TST) or peripheral blood IFNγ release assays (IGRA) such as the T-SPOT.*TB* and QuantiFERON-TB Gold In-Tube. Lower rates of positive TST have been described in elderly populations, compared to younger people [37, 44], including a study of UK nursing home residents that showed a lower rate of TST positivity (3 %) in those aged over 90 years, compared to those under 70 years old (15 %) [45]. However other studies have not shown any age-related difference in TST positivity rate [40]. Studies of repeated TST in the elderly typically show reduced TST reactivity [46, 47], usually interpreted as evidence for waning immunity. Therefore the use of a boosted (two-step) TST is advocated in the elderly to improve sensitivity, though this is not predictive of active TB disease [48].

Data on the use of peripheral blood IGRAs in the elderly are limited. Some suggest indeterminate results may be more likely to occur [49, 50] and other reports show no effect of age [47]. However, discordance between TST and IGRA is more likely to occur in the elderly [47, 51].

There is also controversy in the literature about whether microbiological diagnosis of TB is affected by age, with reports of lower rates in the elderly [52, 53], no difference between groups [54] or higher rates of smear positive sputum in the elderly [40]. There are currently no published reports of the impact of age on GeneXpert molecular diagnostics for TB.

### Age-related effects on anti-tuberculous treatment

Elderly people are more likely to develop adverse drug reactions from polypharmacy, existing co-morbidities and age-related physiological changes. Hepatotoxicity is a common adverse effect as a result of treatment with isoniazid, rifampicin and pyrazinamide, ranging from mild reversible abnormalities of transaminases to fulminant liver failure, and hepatotoxicity from anti-tuberculous drugs rises significantly with increasing age [38, 55, 56]. TB drug related hepatotoxicity in those older than 65 years incurs an odds ratio of 1.71 (CI 95 % 1.24–2.35) [56], although cessation of isoniazid therapy due to suspected hepatotoxicity is not associated with increasing age [57].

Although less common, acute kidney injury is also seen following anti-tuberculous therapy, particularly with rifampicin [58, 59]. This is more likely to occur with increasing age, and with co-morbidities of diabetes mellitus and chronic kidney disease [59].

In addition to increased adverse drug reactions, visual impairment, poor memory and reduced mobility may cause poor adherence to the drug regimen [35].

### Comorbidities and therapies in an ageing population associated with increased risk of TB

Several co-morbidities that are prevalent in ageing populations may further increase the risk of developing active TB disease. Diabetes mellitus increases the risk of active TB by approximately three fold (relative risk 3.11, 95 % CI: 2.27–4.26), in all age groups but may contribute to age related TB as the prevalence of diabetes increases with age [60]. Similarly in chronic obstructive pulmonary disease (COPD), incidence and severity increases with age, and it is also associated with increased rates of active TB, albeit the effect also seems to be independent of age [61].

Furthermore elderly patients are more frequently treated with medication that may suppress protective immunity. The most common example of this is corticosteroids, especially in those with COPD. Both regular inhalers and short courses of high dose corticosteroids during exacerbations are associated with an increased risk of TB in younger and older patients, in a dose-dependent fashion [62–64]. Corticosteroid use (oral prednisolone > 7.5 mg daily) in other diseases is also an independent risk factor for active TB infection [65, 66]. In a study of older people with rheumatic conditions, those on immunosuppressive medication were at an increased risk of mycobacterial infection [67]. Amongst these immunosuppressive therapies, anti-TNF therapy particularly increases the risk of active TB [67–69].

## Conclusions

Given the global ageing population and increasing incidence of active TB in the elderly, it would be prudent to undertake more detailed surveillance of this cohort. Of note there are no data that assess the differential effects of age on transmission of infection, likelihood of developing latent TB or progressing to active disease. Careful evaluation of *de novo* infection and reactivation of LTBI may help to risk stratify patients and target care to those at the highest risk of developing active TB, particularly to assess the role of comorbidities and immunosuppressive treatments. TB in the elderly also generates substantial diagnostic and treatment challenges. The performance of established and novel diagnostics, and new treatment regimens aiming to reduce the duration of treatment in TB should all be specifically evaluated in elderly populations, which have generally been avoided in the past because of practical difficulties in their inclusion and their comorbidities.

### Ethics statement

Ethical approval was not required for this paper as it is a debate and not original research involving humans.

## Abbreviations

CD4: cluster of differentiation 4
CD8: cluster of differentiation 8
COPD: chronic obstructive pulmonary disease
IFNγ: interferon gamma
IGRA: interferon gamma release assay
LTBI: latent tuberculosis infection
Mtb: mycobacterium tuberculosis
TB: tuberculosis
TNF: tumour necrosis factor
TST: tuberculin skin test
UK: United Kingdom
